# Prenatal diagnosis, management, and outcomes of fetuses with tetralogy of Fallot in China after prenatal counseling: a prospective cohort study

**DOI:** 10.3389/fped.2023.1172282

**Published:** 2023-08-09

**Authors:** Deng-pu Deng, Tao Qian, Yu-hong Liu, Zhong-shi Wu, Ting Lu

**Affiliations:** ^1^ Department of Cardiovascular Surgery, The Second Xiangya Hospital of Central South University, Changsha, China; ^2^ National Health Commission Key Laboratory of Birth Defects Research, Prevention, and Treatment, Changsha, China

**Keywords:** tetralogy of Fallot, pregnancy, heart defects, fetal cardiology, fetal echocardiography

## Abstract

**Objective:**

The study aimed to monitor fetuses with tetralogy of Fallot (TOF) after prenatal counseling and how it influenced the decision of parents to terminate the pregnancy.

**Methods:**

Fetuses with isolated TOF diagnosed between January 2019 and December 2021 were prospectively enrolled. The follow-up period extended until termination or 6 months after the operation.

**Results:**

Of the 1,026 fetuses diagnosed with cardiac defects, 129 were identified to have isolated TOF and completed the follow-up. A total of 55 (42.6%) fetuses were terminated, with larger maternal age (odds ratio: 0.893, 95% confidence interval: 0.806–0.989, *P* = 0.031) as the protective factor. The maternal anxiety score, gestational weeks, and pulmonary-to-aortic-diameter ratio lost significance in multivariate analysis. Subjectively, the two most common reasons for terminating the pregnancy were worries about the prognosis (41.8%) and concerns about the possible suffering of the unborn child (18.2%). The prenatal diagnosis was accurate in 73 of the 74 (98.6%) live births. Out of the 64 live births that underwent surgical repair in our center, 57 (89.1%) received primary repair, with a median age of 104 days, and 49 (76.6%) underwent valve-sparing repair. No perioperative death occurred.

**Conclusions:**

Termination for fetuses with TOF remains common in China. Live births with TOF can be safely and effectively managed.

## Introduction

Tetralogy of Fallot (TOF) accounts for 0.28‰ of live births and as much as one-third of all forms of cyanotic congenital heart diseases (CHD) (1–3). The diagnosis of TOF has been largely advanced to the fetal period (4). Tremendous advancements have been made in the management of TOF over the past few decades. The Society of Thoracic Surgeons Congenital Heart Surgery Database report revealed an operative mortality rate of 1.18% between the years 2016 and 2020 (5), which was 1.75% in our center from 2013 to 2020 (6). Long-term survival after TOF repair is encouraging. The earliest prospective cohort study in Erasmus Medical Center demonstrated a cumulative survival rate of 86% at 40 years for living discharges, comparable to that of the general population (7). Strategies to preserve the pulmonary valve and avoid right ventriculotomy, i.e., a transatrial–transpulmonary approach, were expected to improve long-term survival (2).

Fetal detection of TOF offers parents the option of continuing or terminating the pregnancy. With the increase in fetal detection, the rate of termination of pregnancy (ToP) was high in China during the past decade. The Birth Defect Surveillance System in our province revealed 2,838 cases of prenatal diagnosed CHD between 2012 and 2016, 2,476 (87.2%) of which were terminated (8). In addition, 99.6% of fetuses with CHD diagnosed before 28 weeks were terminated (8). Similarly, the ToP rate was 74.7% for all forms of CHD in Zhejiang province (9), 80.4% for TOF in Fujian province (10), and 79.4% for TOF without chromosomal abnormalities in Beijing (11). In these settings, prenatal counseling has been given great attention and performed to help parents of CHD fetuses make sound decisions to continue or terminate the pregnancy.

In our center, the post-counseling ToP rate was 32.4% for all forms of CHD and 44.1% for TOF between the years 2016 and 2018 (12). Thereafter, we started a prospective project to monitor fetuses with CHD following prenatal counseling, which began on 1 January 2019. This study aims to analyze the current pregnancy outcomes and surgical outcomes after prenatal diagnosis of TOF, with a special focus on the determinants for parental decision-making of ToP.

## Materials and methods

Two institutional ethics committees approved the study. Written informed consent to participate in the study and provide truthful information was obtained from the parents.

### Study cohort

Participants were prospectively enrolled in the Second Xiangya Hospital of Central South University and the Maternal and Child Health Hospital of Hunan Province from January 2019 to December 2021. The inclusion criteria included (1) fetuses diagnosed with TOF and (2) parents of the TOF fetuses who signed the informed consent. The exclusion criteria included (1) fetuses with chromosomal abnormalities, genetic mutations, or significant chromosomal microarray analysis abnormalities; (2) fetuses with extracardiac malformations; (3) fetuses with complicated cardiac malformations; and (4) accidental abortion.

### Prenatal diagnosis and counseling

Expert sonographer fellows performed fetal echocardiograms, with pediatric cardiac surgeons reading the reports and obtaining additional images as required.

Both parents were requested to participate in the counseling with a specific pediatric cardiac surgeon (ZW), who mainly provided five types of information as detailed in Supplementary Table S1. Since there is no restriction on ToP for CHD in China, parents were completely autonomous in deciding to continue or terminate the pregnancy. We provided support for the parents but never imposed personal bias into their decision-making. We recommend an invasive genetic diagnosis for all fetuses diagnosed with TOF.

### Data collection and follow-up

Data were collected through pre-counseling questionnaires and post-counseling follow-ups (detailed in Supplementary Table S2). The self-administered pre-counseling questionnaire for the parents investigated the demographic characteristics, the maternal mental health status using the Patient Health Questionnaire-9 (PHQ-9) and Generalized Anxiety Disorder-7 (GAD-7), and the family socioeconomic status (SES). The prenatal follow-up was mainly performed by telephone interview 1 week after the counseling and 1–2 weeks before the expected date of confinement, investigating the quality of counseling, parental pregnancy decision, and subjective reasons for ToP.

Specifically, family SES was calculated according to the household income in the past year and the occupation and education level of each parent, as previously reported in the Chinese CHD cohort (13). The telephone interview involved reading the instructions, questions, and all possible responses verbatim. The quality of counseling was measured 1 week after the counseling in five dimensions on a 5-point Likert scale (Supplementary Table S1). The subjective reasons for ToP were obtained mainly through in-depth interviews with the pregnant women by the counseling expert.

The primary outcome was termination after diagnosis and counseling for fetal TOF, and the secondary outcome was surgery-related mortality for live births (follow-up of up to 6 months after operation). For this study, live births that underwent operations at other institutions were excluded when analyzing the secondary outcomes.

### Statistical analysis

Data were described as mean ± standard deviation, median (interquartile range, IQR) and range, or frequency (%) when appropriate. The differences between the live births and ToP groups were determined by the unpaired Student *t*-test, Mann–Whitney *U*-test, Chi-squared test, or Fisher exact test when appropriate. A univariate logistic regression analysis was performed to identify factors associated with the parental decision of ToP. Variables with *P*-value <0.10 in group comparison or in univariate regression analysis became candidates for multivariate step-backward logistic regression. Results of multivariate regression analysis were reported as a coefficient or odds ratios (OR) with 95% confidence intervals (CI). A *P*-value <0.05 was considered significant. Data analysis was performed using IBM SPSS Statistics 23.0 (SPSS Inc., Chicago, IL, USA).

## Results

### Participants

**Figure 1:**
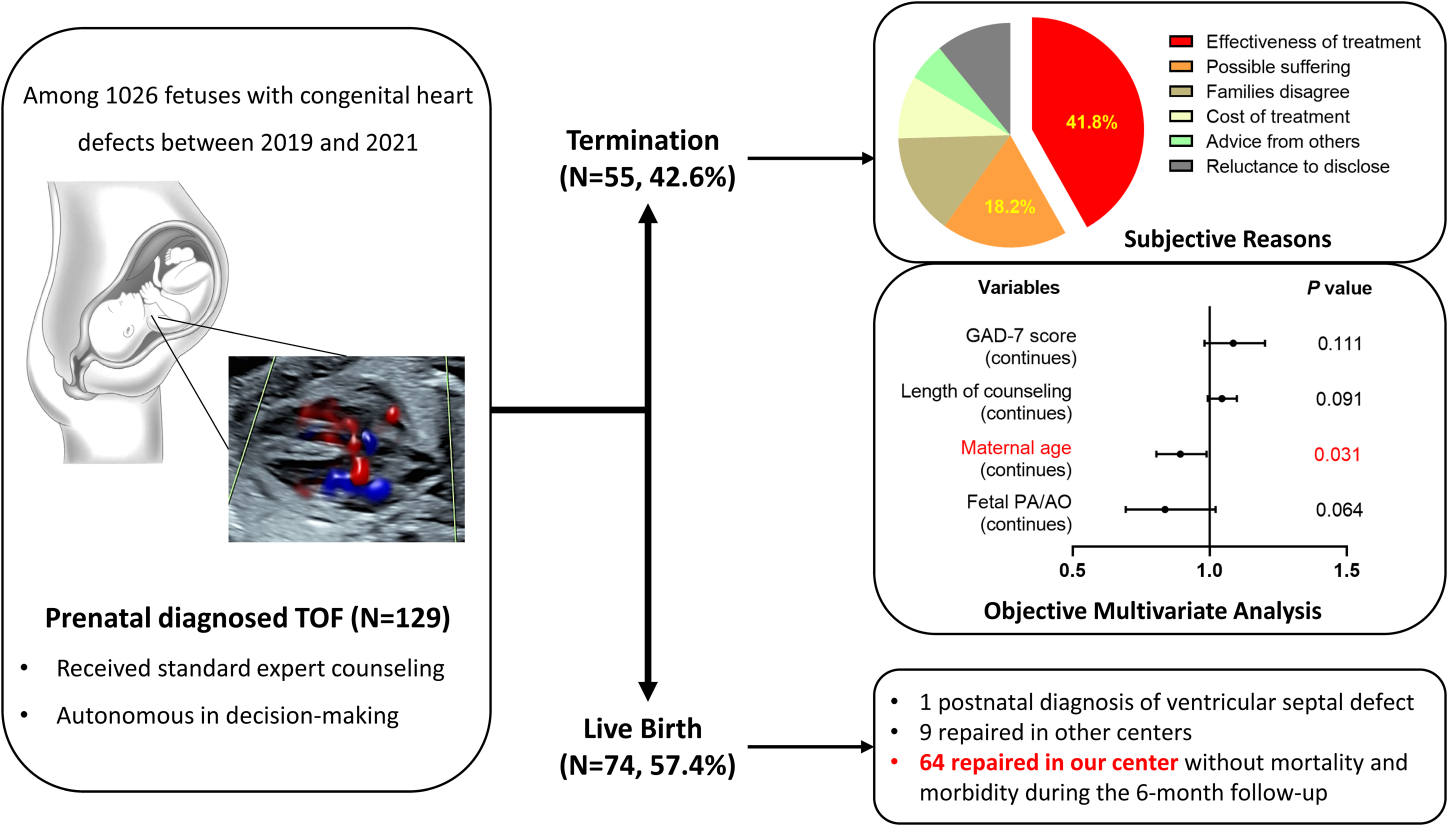
Graphical abstract.

During the study period, 1,026 fetuses were diagnosed with CHD, including 142 cases of TOF. A total of 13 fetuses were excluded according to the pre-set criteria (Supplementary Table S3). Finally, 129 fetuses with isolated TOF were included in the study and completed the follow-up (Figure 1).

Table 1 demonstrates the parental and fetal characteristics categorized by pregnancy outcomes. The average maternal age and paternal age were 30.6 and 33.0 years, respectively. The maternal self-reported PHQ-9 and GAD-7 questionnaires revealed equal or over 10 points in 12 (9.3%) and 19 (14.7%) mothers, respectively, representing significant depression and anxiety before counseling. Only seven (5.4%) families had an annual household income lower than 50,000 yuan/RMB. Family SES was classified into low, medium, and high in 15 (11.6%), 27 (20.9%), and 87 (67.4%) families, respectively (Supplementary Table S4).

**Table 1:** Parental and fetal characteristics for 129 fetuses with tetralogy of Fallot.

	All cohort (*n* = 129)	Live births (*n* = 74)	ToP (*n* = 55)	*P-*value^a^
Maternal characteristics				
Age, continues (years)	30.6 ± 5.0	31.8 ± 5.1	29.1 ± 4.6	0.010
Age >35 years	20 (15.5%)	15 (20.3%)	5 (9.1%)	0.092
The “one-child” policy	25 (19.4%)	16 (21.6%)	9 (16.4%)	0.506
No. of pregnancies				
1	45 (34.9%)	22 (29.7%)	23 (41.8%)	0.192
2	35 (27.1%)	22 (29.7%)	13 (23.6%)	0.549
≥3	49 (38.0%)	30 (40.5%)	19 (34.5%)	0.583
History of childbirth	54 (41.9%)	33 (44.6%)	21 (38.2%)	0.477
History of abortion	61 (47.3%)	37 (50.0%)	24 (43.6%)	0.483
GA at diagnosis (weeks)	23 (22–25)	23 (22–24)	24 (22–25)	0.589
Diagnosis at third trimester	13 (10.1%)	10 (13.5%)	3 (5.5%)	0.153
GA at counseling (weeks)	24 (23–25)	24 (23–26)	24 (23–25)	0.759
Counseling at third trimester	20 (15.5%)	16 (21.6%)	4 (7.3%)	0.029
Length of counseling (mins)	34 ± 9	32 ± 9	36 ± 10	0.080
PHQ-9 score before counseling				
0–4	59 (45.7%)	38 (51.4%)	21 (38.2%)	0.156
5–9	58 (45.0%)	31 (41.9%)	27 (49.1%)	0.476
≥10	12 (9.3%)	5 (6.8%)	7 (12.7%)	0.359
GAD-7 score before counseling				
0–4	62 (48.1%)	44 (59.5%)	18 (32.7%)	0.004
5–9	48 (37.2%)	23 (31.1%)	25 (45.5%)	0.102
≥10	19 (14.7%)	7 (9.5%)	12 (21.8%)	0.077
Paternal and family characteristics				
Paternal age, continues (years)	33.0 ± 5.3	33.9 ± 5.7	31.9 ± 4.7	0.086
Paternal age >35 years	43 (33.3%)	29 (39.2%)	14 (25.5%)	0.131
The “one-child” policy	33 (25.6%)	19 (25.7%)	14 (25.5%)	0.999
Minority family^b^	7 (5.4%)	2 (2.7%)	5 (9.1%)	0.136
Household income (×10,000 yuan/year)	10.2 (8.5–15.0)	11.3 (9.0–16.0)	9.8 (7.8–14.6)	0.462
Family socioeconomic status^c^				
Low	15 (11.6%)	10 (13.5%)	5 (9.1%)	0.581
Middle	27 (20.9%)	14 (18.9%)	13 (23.6%)	0.521
High	87 (67.4%)	50 (67.6%)	37 (67.3%)	0.999
Pregnant and fetal characteristics				
Accidental pregnancy	46 (35.7%)	25 (33.8%)	21 (38.2%)	0.711
Artificial pregnancy	13 (10.1%)	10 (13.5%)	3 (5.5%)	0.153
Twin gestation	12 (9.3%)	10 (13.5%)	2 (3.6%)	0.070
Fetal echo finding				
PA/AO	0.50 ± 0.19	0.55 ± 0.20	0.43 ± 0.16	0.048
VSD/AO	0.67 ± 0.27	0.66 ± 0.28	0.68 ± 0.26	0.827
Absence of ductus arteriosus	9 (7.0%)	5 (6.8%)	4 (7.3%)	0.999

Data are presented as mean ± SD, median (interquartile range), or frequency (%) when appropriate.

^a^ Comparison between the live births and ToP groups via the unpaired Student *t*-test, Mann–Whitney *U*-test, Chi-squared test, or Fisher's exact test when appropriate.

^b^ Minority family was defined as either parent of the fetus or ethnic minorities (non-Han).

^c^ Family socioeconomic status was calculated according to annual household income, parental educational attainment, and employment (detailed in Supplementary Table S3).

The diagnosis of fetal TOF and prenatal counseling were performed during the second trimester for 116 (89.9%) and 109 (84.5%) fetuses, respectively. Meanwhile, both diagnosis and prenatal counseling were performed during the third trimester for the remaining fetuses. The fetal pulmonary-to-aortic-diameter (PA/AO) ratio was 0.50 ± 0.20. The telephone follow-up for the quality of counseling received responses of “agree” or “strongly agree” for all items.

### Pregnancy outcomes

**Figure 2:**
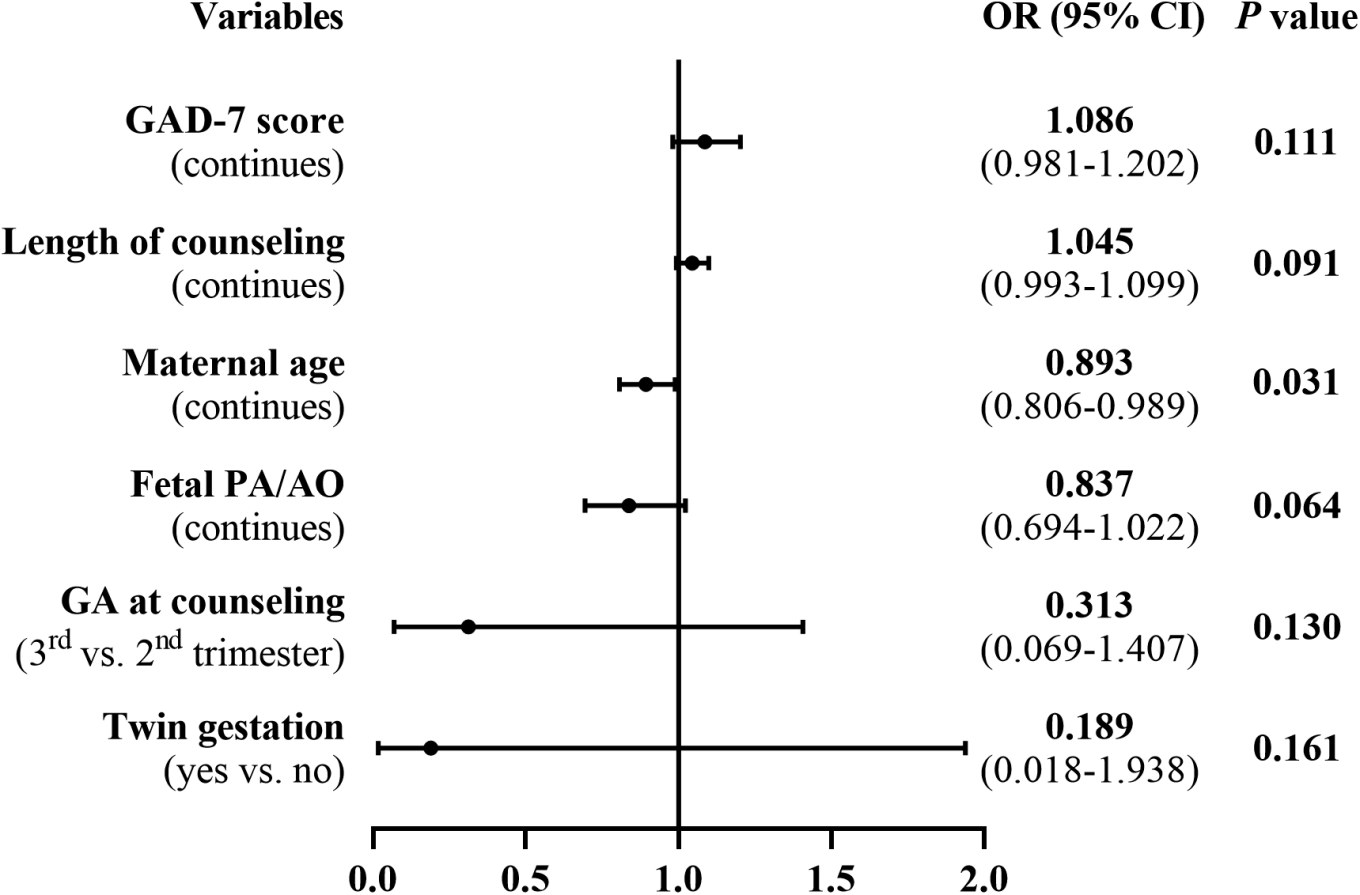
Multivariate analysis of risk factors for parental decision of termination for fetal TOF.

Following prenatal diagnosis and counseling for fetal TOF, there were 55 ToP and 74 live births, resulting in a termination rate of 42.6%. Of the 55 terminations, 36 (65.5%) occurred within 1 week after the counseling and 50 (90.1%) within 2 weeks. A group comparison demonstrated that the maternal age, the proportion of counseling in the third trimester, the proportion of maternal minimal anxiety (GAD-7 score of 0–4), and the PA/AO ratio were significantly larger or higher for live births than those of the ToP group (Table 1). Univariate regression analysis demonstrated the same association between these variables and parental decision of ToP (Supplementary Table S5). On multiple regression analysis, higher maternal age (OR: 0.893, 95% CI: 0.806–0.989, *P* = 0.031) was the only significant protective factor for ToP (Figure 2).

As detailed in Table 2, the most common subjective reason for ToP was worries about the prognosis, accounting for 41.8% of the cases. A total of 10 (18.2%) parents expressed worries about the possible suffering of the unborn child. Only five (9.1%) parents decided to ToP explicitly because they could not afford treatment.

**Table 2:** Subjective reasons for termination of pregnancy for fetuses with tetralogy of Fallot.

Main subjective reasons	Termination of pregnancy (*n* = 55)
(1) Worry about the effectiveness of treatment^a^	23 (41.8%)
(2) Worry about possible suffering of the child^b^	10 (18.2%)
(3) Family members disagree to continue the pregnancy^c^	8 (14.5%)
(4) Worry about the cost of treatment	5 (9.1%)
(5) Advice from other doctors or professionals	3 (5.5%)
(6) Reluctance to disclose	6 (10.9%)

^a^ Parents usually express worries of the unborn child about “not being able to have a normal life after treatment,” “decreased quality of life,” “future number of surgeries,” “exercise limitations,” etc.

^b^ Parents usually express unacceptable that their child “needs surgery within 6 months,” “needs open-chest or open-heart surgery,” etc.

^c^ The mother usually expresses willingness to continue the pregnancy but ultimately decides to terminate after consultation with family members.

Of the 74 live births, 48 (64.9%) were delivered vaginally and 26 (35.1%) required cesarean section for obstetric reasons. The median gestational age (GA) at birth was 38 (IQR: 37–40; range: 30–41) weeks with mean weight of 2.92 ± 0.66 kg (range: 1.70–4.40). Among them, preterm birth occurred in six (8.1%), with median gestational age of 34 (range: 30–36) weeks and mean birth weight of 2.13 ± 0.35 kg.

### Postnatal management

Pre- and postnatal diagnoses were identical in 73 of 74 (98.6%) live births (Supplementary Figure S1). The remaining one fetus was diagnosed with large ventricular septal defect (VSD) postnatally (Supplementary Figure S2). Among the 73 patients with TOF, the median transcutaneous oxygen saturation recorded at newborn screening was 91% (IQR: 87%–94%, range: 72%–97%). Neonatal treatments were recorded in 16 (21.9%) cases, which included 12 cases needing prostaglandin E1 and stent implantation in the right ventricular outflow tract (RVOT) for three cases and in the ductus arteriosus for one case (Table 3).

**Table 3:** Birth information and surgical outcomes for 74 live births with tetralogy of Fallot.

Variables	Live births (*n* = 74)
Male sex (%)	44 (59.5%)
Birth weight (kg)	2.92 ± 0.66
Gestational age at birth (weeks)	38 (37–40)
Postnatal diagnosis	
Large VSD	1 (1.4%)
Tetralogy of Fallot	73 (98.6%)
SpO_2_ (%)	91 (87–94)
Neonatal treatment	16 (21.9%)
Surgical repair of tetralogy of Fallot in our center	(*n* = 64)
Primary repair	57 (89.1%)
Age at repair (days)	104 (85–138)
Weight at repair (kg)	5.83 ± 1.34
Hospitalization costs^a^ (×10,000 yuan/RMB)	8.1 ± 2.0
Staged repair	7 (10.1%)
Age at palliative surgery (days)	18 (7–45)
Age at repair surgery (days)	154 (112–178)
Hospitalization costs^a^ (×10,000 yuan/RMB)	18.5 (15.2–29.4)
Valve-sparing repair	49 (76.6%)
Transannular patch placement	15 (23.4%)
Transatrial VSD closure	64 (100%)
Intraoperative *P*_RV/LV_	0.4 (0.3–0.7)

*P*_RV/LV_, ratio of right ventricular systolic pressure to systolic blood pressure.

Data are presented as mean ± SD, median (interquartile range), or frequency (%) when appropriate.

^a^ Total costs before reimbursing from national medical insurance, which can cover at least 30% of the total cost.

All live births have received surgical treatment, including 64 cases repaired in our center, where primary repair was performed for 57 (89.1%) cases at median age of 104 days (range: 51–176) and mean weight of 5.83 kg (range: 3.4–8.6). Seven (10.9%) patients required staged repair, with three RVOT stenting, two Blalock–Taussig shunts, one ductus arteriosus stenting, and one Sano shunt as the palliative procedures. Finally, 49 (76.6%) patients achieved valve-sparing repair. At the 6-month postoperative follow-up, patients were all alive and free of symptoms.

In our center, the total hospitalization cost for primary TOF repair was 8.1 ± 2.0 × 10,000 yuan/RMB, which was median of 18.5 × 10,000 yuan/RMB (IQR: 15.2–29.4) for staged repair.

## Discussion

We delved into the current proportions and determinants of ToP for fetuses with isolated TOF, as well as surgical outcomes for live births in China, with the cooperation of a large heart center with an annual cardiac surgery volume exceeding 4,000 and a provincial maternal and child healthcare institution. Despite the well-documented safety and efficacy in postnatal management, 42.6% of fetuses diagnosed with TOF were terminated following expert counseling, with younger maternal age and worries about the prognosis identified as the independent risk factor and main subjective reason, respectively.

### Results in the context of what is known

The rate of ToP for CHD was high in China with the increase of fetal detection (8–11). However, during the same period, data mainly from developed countries showed significantly lower rates of termination ranging from 10% to 40%, even in the concomitance of extracardiac or genetic abnormalities (14–18).

Younger maternal age was found significantly associated with a higher rate of ToP, which is reasonable in a cohort with an average pregnant age of 30 years: younger couples usually suggest they had the chance of having another healthy pregnancy and baby. Early gestational age at diagnosis of CHD is a well-proved risk factor for ToP, mainly as the result of less emotional bond between parents and fetuses and less harm to the mother from abortion (19–21). In univariate analysis, we found that gestational age at counseling was more strongly associated with ToP than gestational age at diagnosis, and 90% of ToP happened within 2 weeks after counseling, highlighting the impact of prenatal counseling on parental decision-making. Parental psychological disorders are common after the diagnosis of fetal CHD (22). Significant maternal depression and anxiety in our cohort were less than those in previous reports (23, 24) and were not the risk factors for ToP. Similarly, parental education and occupation levels, family income, and SES were not associated with their decision of ToP in either univariate or multivariate analysis. The most likely explanation is that our study was based on a voluntary counseling cohort, which may be highly biased. Parents with stable emotions and high educational and economic levels were more likely to seek counseling and, therefore, be involved in the cohort. Indeed, 122 (94.8%) families have an annual income sufficient to cover the average hospitalization cost for primary repair of TOF. In comparison, only five (9.1%) families decided to ToP because they could not afford the cost in our cohort.

The accuracy of fetal echocardiography in defining structural heart defects ranged from 70% to 90% (25–27). Herein, only one case of discordance was found between pre- and postnatal anatomical diagnoses in the live births. It was primarily facilitated by our quality control of fetal echocardiography performed by expert sonographers and usually several times to confirm the diagnosis.

Prenatal diagnosis of TOF has a major impact on treating live births, especially neonatal management (4, 28). Typically, we rarely perform repair operations for neonates with TOF. Although repair surgery was the primary strategy (accounting for 60%) for symptomatic neonates in the United States over the past decade (29) and was recommended by many experts (30), it is still a relatively high-risk option for us. More importantly, fetuses with severe pulmonary vascular dysplasia and in high risk for neonatal symptoms were more likely to be terminated, as live births had a significantly higher PA/AO ratio than the ToP group. RVOT stenting was preferred for the changing trend of palliative interventions in the United States for the few unstable neonates (29). In our cohort, three RVOT stenting procedures were performed for severe pulmonary stenosis. Of note, valve-sparing repair was achieved for 76.6% of infants within 6 months. This is, in the current understanding of TOF, the strategy most likely to achieve a satisfactory long-term outcome (2).

### Clinical and research implications

With the increase in the fetal diagnosis of CHD, the option of ToP is more common in a country with liberal interruption laws, especially in the absence of necessary information and support for the parents (31). It is worth noting that the development of a prenatal diagnosis in China was accompanied by the “birth control” and “one-child” policy (32), which is now undergoing major transformation but has resulted in currently low public acceptance of fetal defects. Correspondingly, the most common subjective complaints were worries about the prognosis and the possible suffering of the unborn child. This finding reflects the lack of awareness and the general fear of cardiac defects and explains the significant reduction of the ToP rate after expert counseling compared with the previously reported dramatically high rates in our province (8). It is therefore urgently needed to promote prenatal counseling in clinical practice, as well as strengthen education and scientific popularization to enhance public awareness and acceptance for CHD, in addition to improving the outcomes of surgical treatment.

We point out that pregnancy decision-making is a complex social issue. This study was developed with the primary focus on the relationship between pregnancy outcomes and social environmental factors. We did not yield a statistically significant finding in this regard, probably because of the cohort composition. Future research should investigate the in-depth determinants of parental decision of termination in special situations and social environments.

### Strengths and limitations

Our study is strengthened by its prospective nature, rigorous data quality control, and complete follow-up. In addition, the study included fetuses diagnosed with isolated TOF, thereby eliminating the effects of CHD severity and chromosomal and extracardiac malformations on pregnancy outcomes.

The selection bias of the cohort study largely limited our results. The cohort consisted of voluntary prenatal counseling participants. It is not universal and, therefore, cannot reflect the general outcomes for fetal TOF currently in China but can only represent the best management and outcomes.

## Conclusion

Termination for fetuses with TOF remains common in China. Young women are more likely to decide on ToP. The worries about the prognosis and possible suffering of the unborn child are the main subjective reasons for ToP. Live births with TOF can be safely treated, with very low mortality rates. It is urgently required to promote counseling and strengthen education and scientific popularization to enhance public awareness and acceptance of CHD in the special social environment.

## Data Availability

The original contributions presented in the study are included in the article/Supplementary Material, further inquiries can be directed to the corresponding author.
