# Supplementary material for: Prenatal diagnosis, management, and outcomes of fetuses with tetralogy of Fallot in China after prenatal counseling: a prospective cohort study
Source: Front Pediatr. 2023 Aug 9;11:1172282. doi: 10.3389/fped.2023.1172282 (PMC10445125; doi:10.3389/fped.2023.1172282)
Supplement: Supplementary file 1 [file Datasheet1.docx]

**Table S1. Counseling information for fetuses with cardiac defects and the quality of counseling investigating questionnaire**

| **Counseling information** | | | | | |
| --- | --- | --- | --- | --- | --- |
| 1. Accurate diagnosis of fetal cardiac malformations and its prognostic risk score according to expert consensus^*^ | | | | | |
| 1. Advices on remainder of the pregnancy and perinatal management | | | | | |
| 1. Available treatment options, survival and prognosis analysis | | | | | |
| 1. Emotional and psychological support | | | | | |
| 1. Answers to parental concerns | | | | | |
| **Quality investigation** | | | | | |
|  | Strongly disagree | Disagree | Neutral | Agree | Strongly agree |
| 1. The doctor informed me the diagnosis, management, and prognosis of my fetus |  |  |  |  |  |
| 1. I understood what the doctor was saying |  |  |  |  |  |
| 1. The doctor listened to my questions and concerns |  |  |  |  |  |
| 1. I got psychological comfort and strength |  |  |  |  |  |
| 1. I trust the doctor |  |  |  |  |  |

The counseling information was all provided by a specific pediatric cardiac surgeon. The questionnaire was finished through telephone interview by reading the questions and all possible responses verbatim.

^*^ The Chinese expert consensus (Chinese Journal of Pediatric Surgery, 2018; 39: 163-70+95.), in which imply TOF was scored 3 out of 9.

**Table S2. Information collected for the study**

| **Pre-counseling Questionnaire** | **Post-counseling Follow-ups** |
| --- | --- |
| **Maternal characteristics** | **Other information** |
| Nation and faith | Family address |
| Age (years) | Contact details (Tel., WeChat, e-mail) |
| The only child (yes or no) | Length of counseling (mins) ^†^ |
| Occupation and monthly income (RMB) | **First Follow-up** (1 week after counseling) ^†^ |
| Education level * | Parental decision (ToP or not) |
| Number of pregnancies and births | Gestational age at ToP (weeks) |
| PHQ-9 and GAD-7 score | Subjective reasons for ToP |
| **Paternal and family characteristics** | Quality of the counseling (1-5) ^‡^ |
| Race and faith | **Second Follow-up** (1-2 weeks before EDC) ^†^ |
| Paternal age (years) | Parental decision (ToP or not) |
| The only child (yes or no) | Accidental miscarriage (yes or no) |
| Occupation and monthly income (RMB) | Birth plan (when, where, and how) |
| Education level ^*^ | **Postnatal Follow-up** ^†^ |
| Other children (yes or no) | Date of birth (date) |
| Gender, age, and health status of other children | Gestational age at birth (weeks) |
| **Pregnant and fetal characteristics** | Gender (female or male) |
| Accidental pregnancy (yes or no) | Weight at birth (kg) |
| Artificial pregnancy (yes or no) | Cardiac diagnosis |
| Twin gestation (yes or no) | Neonatal treatment |
| Gestational age at diagnosis and counseling (weeks) | Date of surgery (date) |
| Expected date of confinement (date) | Weight at surgery (kg) |
| Fetal cardiac diagnosis and prognostic risk score ^†^ | Surgical institution and strategies ^§^ |
| Combined malformations or abnormalities ^†^ | Hospitalization costs (RMB) |
| Therapeutic planning | Outcomes |

EDC, expected date of confinement; GAD-7, Generalized Anxiety Disorder-7; PHQ-9, Patient Health Questionnaire-9; ToP, termination of the pregnancy.

^*^ Education level was categorized as less than high school, high school graduate or equivalent, college graduate or above.

^†^ Information recorded by research assistants or cardiac surgeons.

^‡^ The responses were measured in 5 dimensions and scored on a five-point Likert scale as shown in Table S2.

^§^ Surgical strategies for patients with tetralogy of Fallot included primary repair or staged repair, valve-sparing repair or transannular patch placement.

**Table S3. Reasons and** **pregnant outcomes for 13 excluded cases**

| **Excluding Reasons** | **Numbers** | **Pregnant Outcomes** |
| --- | --- | --- |
| **Genetic abnormalities** ^*^ |  |  |
| 22q11.2 microdeletion | 4 | 3 ToP; 1 live birth |
| Trisomy 21 | 1 | ToP |
| 1q21.1 microduplication | 1 | ToP |
| **Extracardiac malformations** |  |  |
| Single kidney | 1 | ToP |
| Short long bone | 1 | ToP |
| Fetal growth restriction | 1 | ToP |
| Cerebellar hypoplasia | 1 | ToP |
| **Complicated cardiac malformations** |  |  |
| Complete atrioventricular septal defect | 1 | Live birth |
| Left pulmonary artery sling | 1 | Live birth |
| **Accidental abortion** | 1 | Unexpected ToP |

ToP, termination of the pregnancy

^*^ Invasive genetic diagnosis (amniotic fluid or cord blood analysis) was performed in 86 of 142 (60.5%) fetuses with tetralogy of Fallot.

**Table S4.** **Socioeconomic status of 129 families with fetuses diagnosed with TOF**

|  | **Family Socioeconomic Status (n=129)** | | |
| --- | --- | --- | --- |
|  | Low (n=15) | Medium (n=27) | High (n=87) |
| Paternal education ^*^ |  |  |  |
| Low | 12 (80.0%) | 7 (25.9%) | 0 |
| Medium | 3 (20.0%) | 16 (59.3%) | 16 (18.4%) |
| High | 0 | 4 (14.8%) | 71 (81.6%) |
| Maternal education ^*^ |  |  |  |
| Low | 15 (100.0%) | 6 (22.2%) | 0 |
| Medium | 0 | 18 (66.7%) | 15 (17.2%) |
| High | 0 | 3 (11.1%) | 72 (82.8%) |
| Paternal occupation ^†^ |  |  |  |
| Low | 15 (100.0%) | 9 (33.3%) | 3 (3.5%) |
| Medium | 0 | 17 (63.0%) | 59 (67.8%) |
| High | 0 | 1 (3.7%) | 25 (28.7%) |
| Maternal occupation ^†^ |  |  |  |
| Low | 8 (53.3%) | 16 (59.3%) | 9 (10.4%) |
| Medium | 7 (46.7%) | 11 (40.7%) | 43 (49.4%) |
| High | 0 | 0 | 35 (40.2%) |
| Annual household income ^‡^ |  |  |  |
| Low | 3 (20.0%) | 3 (11.1%) | 1 (1.2%) |
| Medium-low | 10 (66.7%) | 20 (74.1%) | 27 (31.0%) |
| Medium | 2 (13.3%) | 4 (14.8%) | 28 (32.2%) |
| Medium-high | 0 | 0 | 21 (24.1%) |
| High | 0 | 0 | 10 (11.5%) |

Family socioeconomic status is measured as previous reported in Chinese cohort of congenital heart diseases by Xiang Li *et.al.* (J Am Heart Assoc. 2019 Jan 8;8(1): e010616).

^*^ Education was categorized as less than high school (low), high school graduate or equivalent (medium), and college graduate or above (high).

^†^ Occupation was categorized as manual worker, farmer, or unemployed (low); businessman or clerk (medium); and professional, manager, or government employee (high).

^‡^ Annual household income was categorized as low (<¥50 thousand), medium-low (¥50-100 thousand), medium (¥100-150 thousand), medium-high (¥150–200 thousand), and high (≥¥200 thousand).

**Table S5. Univariate logistics regression analysis of variables associated with termination of the pregnancy following fetal diagnosis of TOF**

|  | ***P* value** |
| --- | --- |
| **Maternal characteristics** |  |
| Age (continues) | 0.013 |
| Age >35 years (yes vs. no) | 0.218 |
| The “only child” (yes vs. no) | 0.433 |
| Occupations (category) | 0.417 |
| Education level (category) | 0.554 |
| First pregnancy (yes vs. no) | 0.280 |
| Primipara (yes vs. no) | 0.599 |
| History of abortion (yes vs. no) | 0.548 |
| PHQ-9 score (continues) | 0.113 |
| PHQ-9 score >10 (yes vs. no) | 0.275 |
| GAD-7 score (continues) | 0.043 |
| GAD-7 score >10 (yes vs. no) | 0.097 |
| **Paternal and family characteristics** |  |
| Paternal age (continues) | 0.089 |
| Paternal age >35 years (yes vs. no) | 0.158 |
| Parental age gap (continues) | 0.972 |
| The “only child” (yes vs. no) | 0.969 |
| Both “only child” (yes vs. no) | 0.136 |
| Minority family (yes vs. no) | 0.131 |
| Paternal occupations (category) | 0.555 |
| Paternal education level (category) | 0.423 |
| Annual household income (category) | 0.897 |
| Family SES level (category) | 0.922 |
| **Pregnant and fetal characteristics** |  |
| Accidental pregnancy (yes vs. no) | 0.664 |
| Artificial pregnancy (yes vs. no) | 0.185 |
| Twin gestation (yes vs. no) | 0.095 |
| GA at diagnosis (continues) | 0.598 |
| GA at diagnosis (3^rd^ vs. 2^nd^ trimester) | 0.185 |
| GA at counseling (continues) | 0.268 |
| GA at counseling (3^rd^ vs. 2^nd^ trimester) | 0.078 |
| Length of counseling (continues) | 0.084 |
| Fetal PA/AO (continues) | 0.040 |
| Fetal VSD/AO (continues) | 0.827 |

GAD-7, Generalized Anxiety Disorder-7; GA, gestational age; PA/AO, ratio of the pulmonary and aortic diameter; PHQ-9, Patient Health Questionnaire-9; SES, socioeconomic status; VSD/AO, ratio of ventricular septal defect and aortic diameter.
