# Supplementary figures and images for: Prenatal diagnosis, management, and outcomes of fetuses with tetralogy of Fallot in China after prenatal counseling: a prospective cohort study
Source: Front Pediatr. 2023 Aug 9;11:1172282. doi: 10.3389/fped.2023.1172282 (PMC10445125; doi:10.3389/fped.2023.1172282)

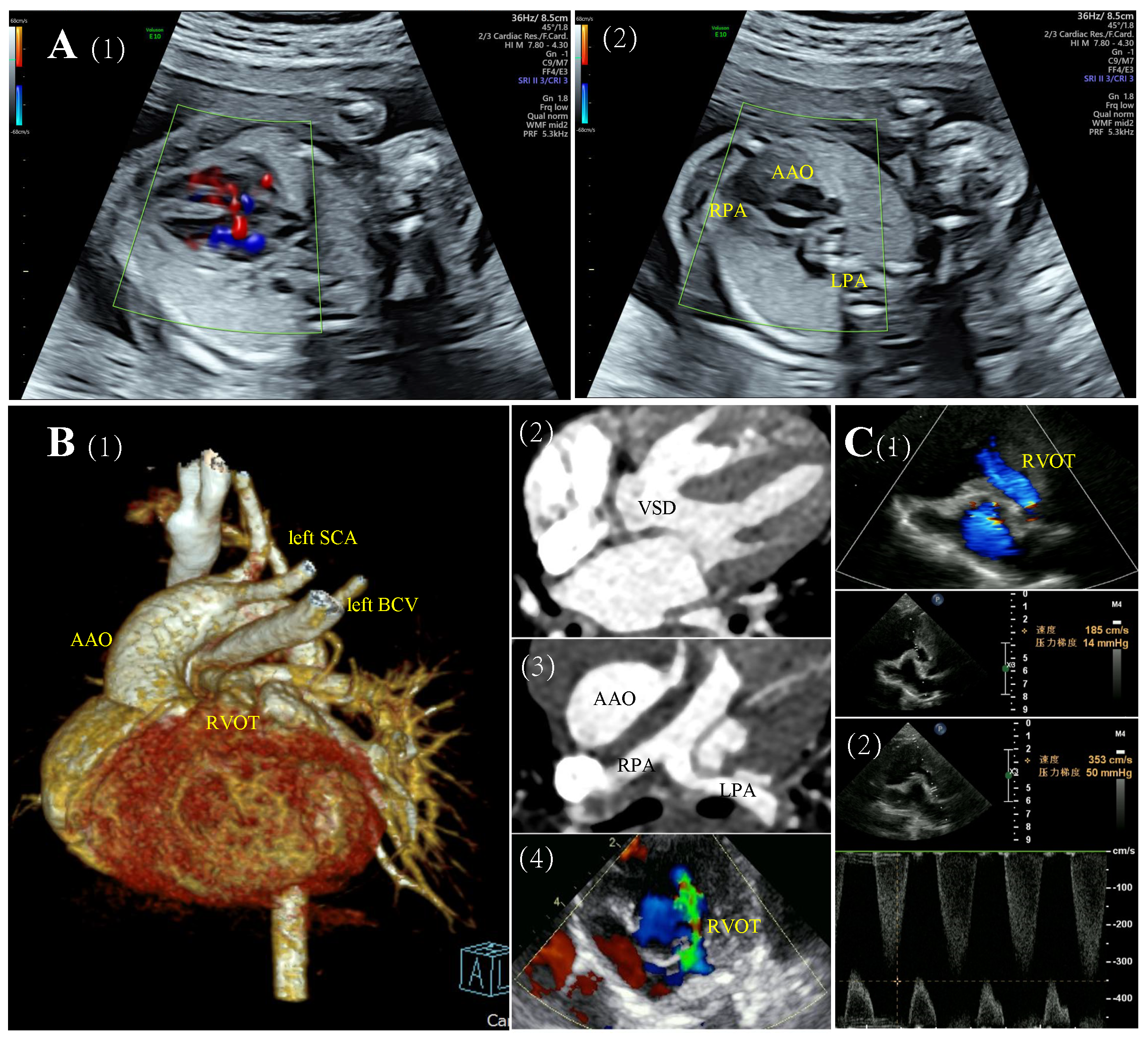

Supplement: Supplementary file 2 [file Image1.tif]

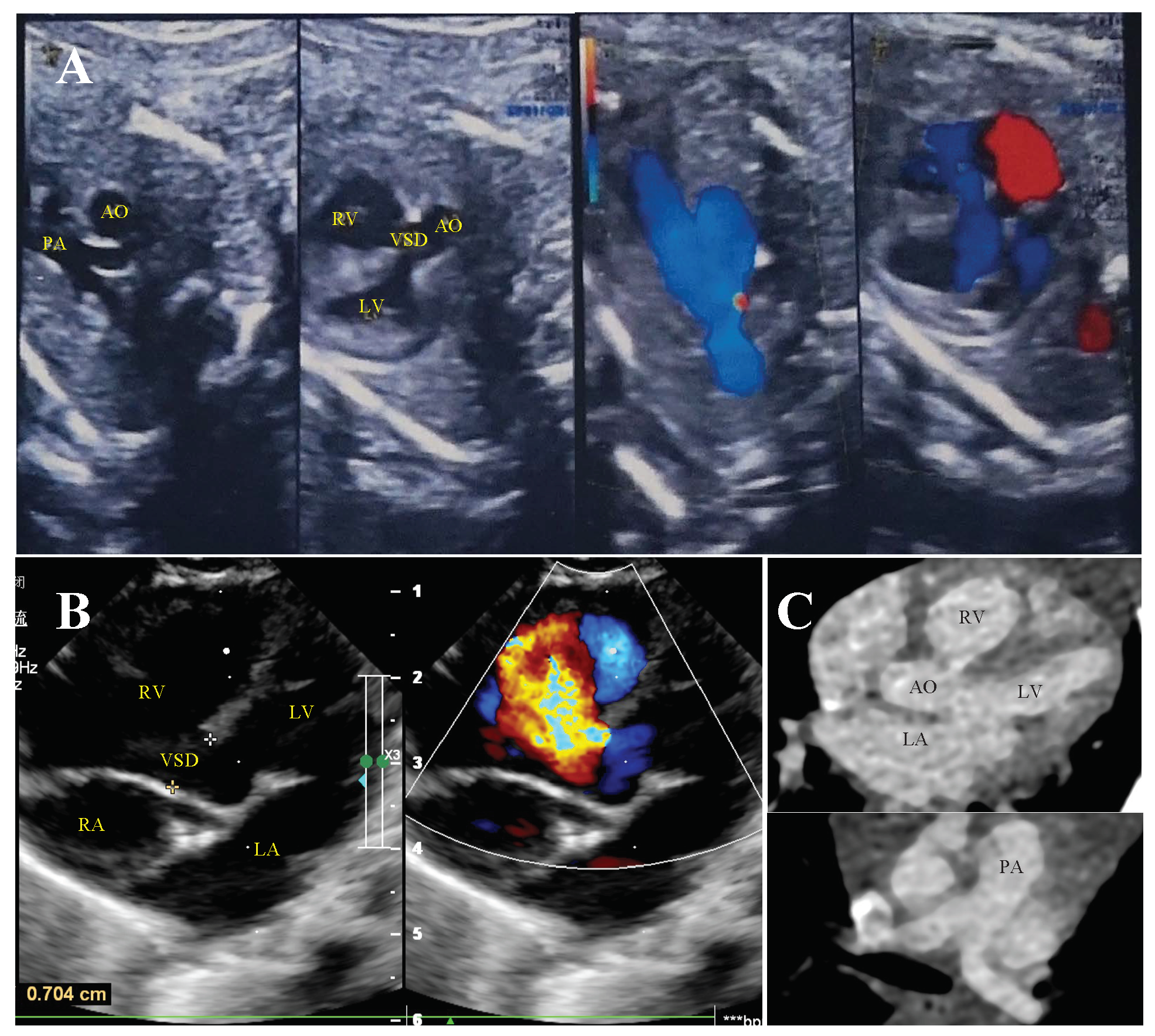

Supplement: Supplementary file 3 [file Image2.tif]
